# Accuracy of Wristband Fitbit Models in Assessing Sleep: Systematic Review and Meta-Analysis

**DOI:** 10.2196/16273

**Published:** 2019-11-28

**Authors:** Shahab Haghayegh, Sepideh Khoshnevis, Michael H Smolensky, Kenneth R Diller, Richard J Castriotta

**Affiliations:** 1 Department of Biomedical Engineering Cockrell School of Engineering The University of Texas at Austin Austin, TX United States; 2 Division of Pulmonary and Sleep Medicine, Department of Internal Medicine McGovern School of Medicine The University of Texas Health Science Center at Houston Houston, TX United States; 3 Division of Pulmonary, Critical Care and Sleep Medicine Keck School of Medicine University of Southern California Los Angeles, CA United States

**Keywords:** Fitbit, polysomnography, sleep tracker, wearable, actigraphy, sleep diary, sleep stages, accuracy, validation, comparison of performance

## Abstract

**Background:**

Wearable sleep monitors are of high interest to consumers and researchers because of their ability to provide estimation of sleep patterns in free-living conditions in a cost-efficient way.

**Objective:**

We conducted a systematic review of publications reporting on the performance of wristband *Fitbit* models in assessing sleep parameters and stages.

**Methods:**

In adherence with the Preferred Reporting Items for Systematic Reviews and Meta-Analyses (PRISMA) statement, we comprehensively searched the Cumulative Index to Nursing and Allied Health Literature (CINAHL), Cochrane, Embase, MEDLINE, PubMed, PsycINFO, and Web of Science databases using the keyword *Fitbit* to identify relevant publications meeting predefined inclusion and exclusion criteria.

**Results:**

The search yielded 3085 candidate articles. After eliminating duplicates and in compliance with inclusion and exclusion criteria, 22 articles qualified for systematic review, with 8 providing quantitative data for meta-analysis. In reference to polysomnography (PSG), nonsleep-staging *Fitbit* models tended to overestimate total sleep time (TST; range from approximately 7 to 67 mins; effect size=-0.51, *P*<.001; heterogenicity: I^2^=8.8%, *P*=.36) and sleep efficiency (SE; range from approximately 2% to 15%; effect size=-0.74, *P*<.001; heterogenicity: I^2^=24.0%, *P*=.25), and underestimate wake after sleep onset (WASO; range from approximately 6 to 44 mins; effect size=0.60, *P*<.001; heterogenicity: I^2^=0%, *P*=.92) and there was no significant difference in sleep onset latency (SOL; *P*=.37; heterogenicity: I^2^=0%, *P*=.92). In reference to PSG, nonsleep-staging *Fitbit* models correctly identified sleep epochs with accuracy values between 0.81 and 0.91, sensitivity values between 0.87 and 0.99, and specificity values between 0.10 and 0.52. Recent-generation *Fitbit* models that collectively utilize heart rate variability and body movement to assess sleep stages performed better than early-generation nonsleep-staging ones that utilize only body movement. Sleep-staging *Fitbit* models, in comparison to PSG, showed no significant difference in measured values of WASO (*P*=.25; heterogenicity: I^2^=0%, *P*=.92), TST (*P*=.29; heterogenicity: I^2^=0%, *P*=.98), and SE (*P*=.19) but they underestimated SOL (*P*=.03; heterogenicity: I^2^=0%, *P*=.66). Sleep-staging *Fitbit* models showed higher sensitivity (0.95-0.96) and specificity (0.58-0.69) values in detecting sleep epochs than nonsleep-staging models and those reported in the literature for regular wrist actigraphy.

**Conclusions:**

Sleep-staging *Fitbit* models showed promising performance, especially in differentiating wake from sleep. However, although these models are a convenient and economical means for consumers to obtain gross estimates of sleep parameters and time spent in sleep stages, they are of limited specificity and are not a substitute for PSG.

## Introduction

Polysomnography (PSG) consists of simultaneous electroencephalographic (EEG), electromyographic, electrooculographic, electrocardiographic, and other assessments. PSG is regarded as the gold standard for diagnosis of sleep disorders and conduct of sleep research. However, the environment and instrumentation of conventional PSG can be uncomfortable, anxiety producing, and even sleep disturbing. Additionally, PSG requires a special facility plus oversight by skilled technicians, making it expensive and precluding, under most circumstances, investigation of between-night variation of sleep quality. Thus, it is not surprising less than 50% of sleep studies nowadays are conducted in formal sleep facilities [1].

Sleep diary methods are simple and economical ways of tracking and appraising sleep by consumers but because they entail subjective self-ratings, they are often inaccurate and incomplete; furthermore, they do not assess sleep architecture and stages. EEG wearables enable at-home evaluation of sleep architecture and staging but they are expensive and somewhat technologically complicated. Wrist actigraphy, which senses accelerated motion, was introduced some 35 years ago by Ambulatory Monitoring Inc and is now used in conjunction with proprietary interpretative algorithms to conduct outpatient sleep screenings through estimation of key sleep parameters. Nonetheless, these devices, which rely entirely on movement-based algorithms [2], lack sleep-stage assessment capability; additionally, they tend to overestimate sleep duration [3]. Approximately 10 years ago, *Fitbit* (Fitbit, Inc) introduced its first wearable model [4] for use by health-conscious consumers. Early-generation Fitbit models only determined sleep parameters. However, subsequent modifications and refinements, including a scoring algorithm based collectively on body movement and heart rate variability (HRV), enable recent-generation Fitbit models—Fitbit Charge 2, Fitbit Charge 3, Fitbit Alta HR, Fitbit Versa, Fitbit Versa 2, Fitbit Blaze, Fitbit Inspire HR, and Fitbit Ionic—to estimate not only sleep parameters and stages [5], but wake- and sleep-time heart rate [6]. A 2019 survey found wearable technology to be the number one fitness trend worldwide [7]. Fitbit wearables, in particular, are very popular among consumers, with more than 25 million active users in more than 80 countries [8]. Additionally, they are the most-used wearables for conducting biomedical research [9]. In this regard, this year the United States National Institutes of Health announced its decision to incorporate Fitbit technology into its *All of Us Research Program* [10,11]. Nonetheless, the accuracy of Fitbit technology remains a major concern, not only of medical professionals but the lay public [12]. In recognition of the growing interest and use of personal wristband devices to routinely self-assess biomarkers of sleep quality, the National Sleep Foundation, the Consumer Technology Association, and the American National Standards Institute developed recommended terminology and definitions to describe sleep features derived by such products [13]. Given the growing popularity with consumers and medical organizations of the Fitbit wristband devices, the objective of this paper is to appraise, in compliance with these recommendations, the performance of both early- and recent-generation Fitbit models in determining sleep parameters and sleep stages through a systematic review of findings of relevant publications and a meta-analysis of reported data.

## Methods

This prospective systematic review, which was not registered beforehand, was conducted in accordance with the Preferred Reporting Items for Systematic Reviews and Meta-Analyses (PRISMA) statement [14].

### Search Strategy

An online comprehensive search of the following databases was performed: the Cumulative Index to Nursing and Allied Health Literature (CINAHL), the Cochrane Database, Embase, MEDLINE, PsycINFO, PubMed, and Web of Science. The search was initially performed during July 2018 and again during July and October 2019 using the keyword *Fitbit* without language, publication date, or other filters.

### Eligibility Criteria

Retrieved publications qualified for the systematic review if they (1) involved validity of sleep data of any marketed Fitbit model and (2) incorporated PSG, actigraphy, home EEG, sleep diary, or survey method as reference. Exclusion criteria included the following: (1) sample size of less than 5 participants, (2) review paper, (3) absent or inappropriate statistical analysis, and (4) duplicate publication of the same data and findings.

### Study Selection

Citations were imported into the reference manager software Mendeley. After elimination of duplicate reports, one author (SH) screened titles and abstracts first to remove unrelated publications; thereafter, two authors (SH and SK) independently screened remaining publications for eligibility according to inclusion and exclusion criteria. Disagreements were resolved by discussion.

### Data Extraction and Items

The following items were extracted in a systematic manner by one author (SH) and checked for accuracy by another author (SK): first author; year of publication; type of sleep tracker and comparator; number, sex, type, and age of participants; study site; number of nights of sleep assessment; bedtime; Fitbit mode setting; anatomical placement of tracker; and study outcomes relative to the denoted reference standard—the precision of measuring the parameters of total sleep time (TST), sleep onset latency (SOL), wake after sleep onset (WASO), and sleep efficiency (SE), as well as the sensitivity, specificity, and accuracy of detecting both sleep epochs and sleep stages.

### Bias Assessment

A checklist, adapted from Downs and Black [15], was applied to evaluate each publication for quality and risk of bias of research methods, internal validity, reported outcomes, and generalizability.

### Statistical Analysis

For studies that compared Fitbit with PSG and provided quantitative data, raw Hedges g effect sizes of SOL, WASO, TST, and SE were calculated as the mean differences between average values provided by Fitbit and PSG divided by the standard deviation of PSG values multiplied by the Hedges correction factor [16]. A positive effect size infers lower values derived by Fitbit relative to those derived by PSG. Overall effect size and 95% prediction interval for each parameter per nonsleep-staging and sleep-staging Fitbit models were calculated using a random-effects model [17,18]. Forest plots were created by Microsoft Excel for Mac, version 16.25 (Microsoft Corporation). A threshold probability of 5% (*P*=.05) was selected as the basis for rejecting the null hypothesis, effect size equals zero. Effect size values of 0.2, 0.5, and 0.8 are considered small, medium, and large effects, respectively [19]. The null hypothesis stating that studies share a common effect size per sleep parameter was tested by calculating the Q statistic [17]. τ^2^ represents the overall variation of true effect size, and I^2^ represents the proportion of observed variance indicative of actual variation among studies [17]. I^2^ values in the order of 25%, 50%, and 75% are considered small, medium, and large heterogenicity, respectively [20]. Comparisons between nonsleep-staging and sleep-staging Fitbit models were accomplished by random-effect subgroup analyses. As recommended, the threshold probability of 10% (*P*=.10) was the basis for testing the significance of heterogenicity and also for determining statistical significance of subgroup comparisons [20,21].

## Results

### Search Results

**Figure 1:**
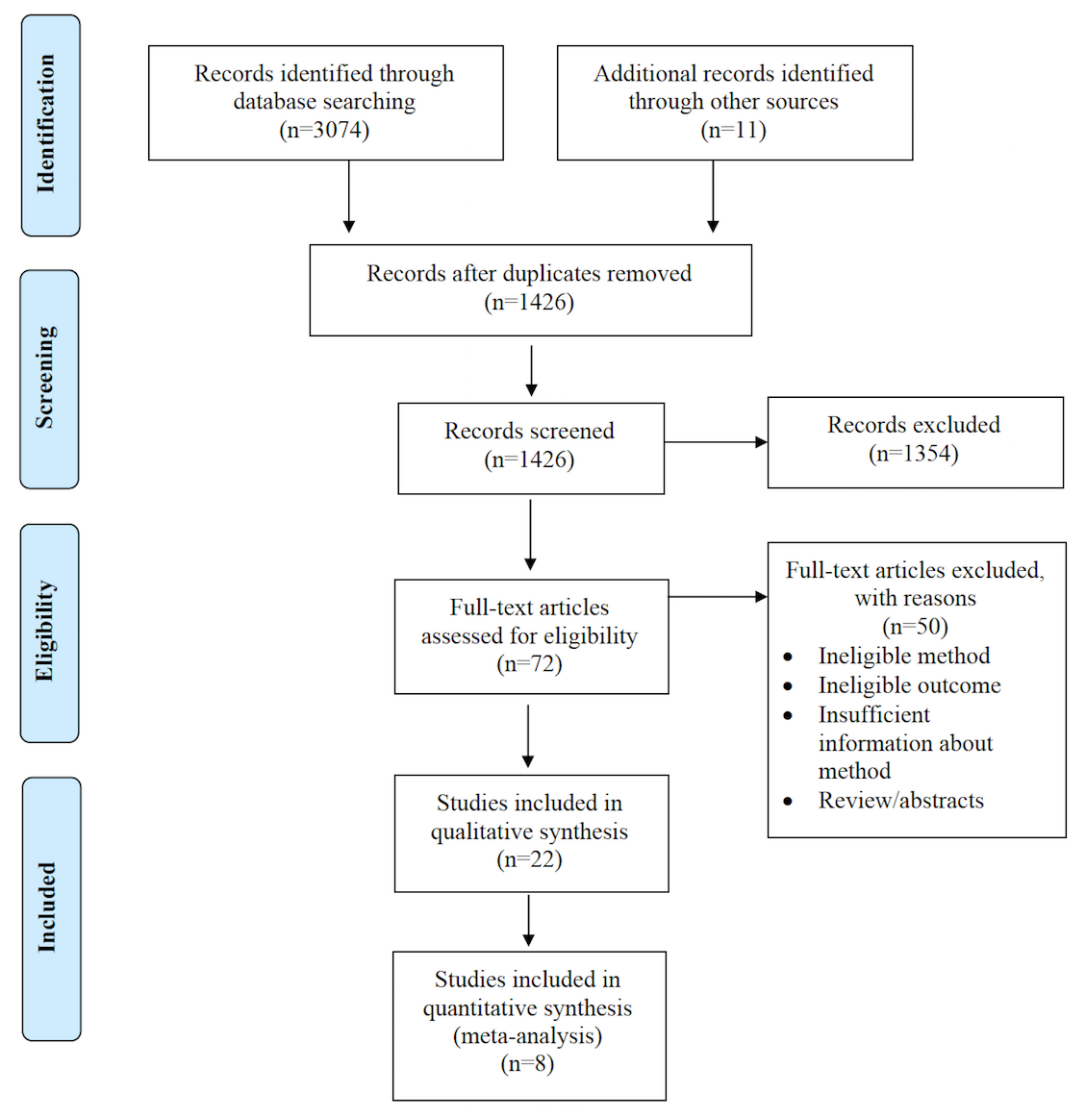
Flow diagram adapted from Moher et al [14] describing the search strategy of databases to retrieve and qualify publications of relevance for review.

Figure 1 presents a visual summary of the selection and qualification of articles for review. A total of 3074 publications were retrieved though a search of databases performed in July 2018 and again in July and October 2019. An additional 11 publications were identified through other sources, primarily through the reference list of identified articles. After eliminating duplicate publications found in the multiple databases, 1426 articles remained for screening. Examination of individual titles and abstracts yielded 72 publications for full-text appraisal; however, after scrutinizing each of them according to the a priori inclusion and exclusion criteria, only 22 qualified for systematic review [22-43], with 8 of ﻿these reporting raw data to enable quantitative synthesis and meta-analyses.

### Overview of Included Studies

Tables 1 and 2 present the extracted details of each qualifying study involving nonsleep-staging and sleep-staging Fitbit models. Participants were diverse: normal sleepers as well as persons diagnosed with periodic limb movement in sleep (PLMS) [28], obstructive sleep apnea, sleep-disordered breathing [30], central disorders of hypersomnolence [26], insomnia [31], and depression [25], and Huntington disease gene carriers [38]. Sample size varied substantially between investigations, from 7 to 63 (median 30) participants, with approximately 77% of them involving more than 20 individuals. Average age was less than 20 years in 6 of the 22 studies (27%) and over 50 years in 1 study (5%). Out of 22 studies, 10 (45%) were conducted in a sleep laboratory, 11 (50%) in the home environment, and 1 (5%) either at home or in a hotel, based on the participant’s preference.

**Table 1:** Detailed summary of qualifying publications involving both early-generation, nonsleep-staging and newer-generation, sleep-staging Fitbit models.

Author (year)	Model	Reference	Participants			Investigative details		
			Total number (% female)	Age (years), mean (SD); and/or range	Type	Duration (study site)	Tracker placement (tracker mode)	Bedtime
Beattie et al (2017) [22]^a^	Surge	Type III home PSG^b^	60 (40.0)	34 (10)	Normal sleepers	1 night (home or hotel)	Both left and right wrists (N/A^c^)	22:00h
Brazendale et al (2019) [23]	Charge HR	Sleep log & actigraphy	30 (37.0)	7.2 (2.1)	Healthy	2 nights (home)	Nondominant wrist (N/R^d^)	Habitual^e^
Brooke et al (2017) [24]	Flex & Charge HR	Sleep log	95 (64.2): 22 Flex; 14 Charge HR	28.5 (9.9); 19-60	Healthy	1 night (home)	Left wrist (N/R)	Habitual^e^
Cook et al (2017) [25]	Flex	PSG & actigraphy	21 (81.0)	26.5 (4.6)	Major depressive disorder	1 night (sleep lab)	Nondominant wrist (both sensitive and normal)	Habitual
Cook et al (2019) [26]^a^	Alta HR	PSG	49 (93.9)	30.3 (9.8)	Suspected CDH^f^	1 night (sleep lab)	Nondominant wrist (N/A)	Participant preference
de Zambotti et al (2016) [27]	Charge HR	PSG	32 (46.9)	17.3 (2.5); 12-21	Healthy adolescents	1 night (sleep lab)	Nondominant wrist (normal)	Participant preference
de Zambotti et al (2018) [28]^a^	Charge 2	PSG	44 (59.1)	19-61	Healthy adults, 9 with PLMS^g^	1 night (sleep lab)	Nondominant wrist (N/A)	Participant preference
Dickinson et al (2016) [29]	Charge HR	Actigraphy	38 (60.5)	26.1 (8.0)	Young adults	4 nights (home)	N/R (normal)	Habitual^e^
Hakim et al (2018) [30]	Charge HR	PSG	22 (59.1)	9 (3); 3-18	OSA^h^ or SDB^i^	1 night (sleep lab)	N/R (N/R)	N/R
Kang et al (2017) [31]	Flex	Unattended PSG	Insomniacs: 33 (57.6); good sleepers: 17 (64.7)	Insomniacs: 38.4 (11.2); good sleepers: 32.1 (7.4)	Insomniacs and good sleepers	1 night (home)	Nondominant wrist (both sensitive and normal)	N/R
Kubala et al (2019) [32]	Alta	Actigraphy	Good sleepers: 20 (60); poor sleepers 10 (30)	24.8 (4.1); 18-60	Healthy adults	7 nights: Fitbit was worn for only 1 night (home)	Nondominant wrist (normal)	Habitual^e^
Lee et al (2017) [33]	Charge HR	Actigraphy	16 (62.5)	22.8 (2.8); 18-26	Healthy young adults	13 nights (home)	Nondominant hand (normal)	Habitual^e^
Lee et al (2018) [34]	Charge HR	Sleep log	38 (50)	28.6 (N/R); 19-66	Healthy	3 nights (home)	Counter-balanced across participants (N/R)	Habitual^e^
Liang and Chapa Martell (2018) [35]	Charge 2	Sleep Scope (EEG^j^ based)	25 (40.0)	24.8 (4.4)	Healthy	3 nights: only 1 night in data analysis (home)	Nondominant hand (normal)	Habitual^e^
Liu et al (2019) [36]^a^	Alta HR	Sleep log	10 (50.0)	21.8 (N/R); 20-24	Noninsomniac Asian students	7 nights (home)	Left wrist (N/A)	Habitual^e^
Mantua et al (2016) [37]	Flex	PSG (ambulatory system)	40 (47.5)	22.4 (4.9); 18-30	Healthy young adults	1 night (home)	Counter-balanced across participants (N/R)	N/R
Maskevich et al (2017) [38]	One	PSG	7 (85.7)	54.1 (6.4)	Huntington disease gene carriers	1 night (sleep lab)	Nondominant wrist (default)	Habitual
Meltzer et al (2015) [39]	Ultra	PSG & actigraphy (2 different ones)	63 (50.8)	9.7 (4.6); 3-17	Children and adolescents	1 night (sleep lab)	Nondominant wrist (both sensitive and normal)	Habitual
Montgomery-Downs et al (2012) [40]	Classic	PSG & actigraphy	24 (40)	26.1 (N/R); 19-41	Healthy adults	1 night (sleep lab)	Nondominant wrist (N/R)	N/R
Osterbauer et al (2016) [41]	Flex	PSG	14 (64.3)	6.5 (2.9); 3-11	Children	1 night (sleep lab)	Nondominant wrist (N/R)	N/R
Sargent et al (2018) [42]	Charge HR	PSG	12 (N/R)	18.3 (1.0)	Soccer players without sleep disorders	5 sleep periods during 3 days and nights (sleep lab)	Nondominant wrist (normal)	22:00-07:00h); 23:00-07:00h); 00:00-07:00h); 14:00-16:00h); 15:00-16:00h
Svensson et al (2019) [43]^a^	Versa	Sleep Scope (EEG based)	20 (50)	25-67	Healthy Japanese adults	14 nights: only 7 nights in data analysis (home)	Nondominant wrist (normal)	Habitual^e^

^a^Publication consisting of newer-generation, sleep-staging Fitbit models.

^b^PSG: polysomnography.

^c^N/A: not applicable.

^d^N/R: not reported.

^e^Exact bedtime not reported, but investigative protocol infers habitual bedtime.

^f^CDH: central disorders of hypersomnolence.

^g^PLMS: periodic limb movement in sleep.

^h^OSA: obstructive sleep apnea.

^i^SDB: sleep-disordered breathing.

^j^EEG: electroencephalographic.

**Table 2:** Results from qualifying publications involving both early-generation, nonsleep-staging and newer-generation, sleep-staging Fitbit models.

Author (year)	Model	Reference	Results^a^
Beattie et al (2017) [22]^b^	Surge	Type III home PSG^c^	Normal mode vs PSG. Overestimated TST^d,e^ (46 min) and SE^e,g^ (8.1%); underestimated WASO^e,h^ (44 min) and ﻿SOL^i^ (2 min, NS^j^); accuracy, 0.88 (SD 0.05); sensitivity, 0.98 (SD 0.02); specificity, 0.35 (SD 0.13) Normal mode vs actigraphy. Overestimated SOL^e^ (12 min), SE^f^ (1.1%), and TST (5 min, NS); underestimated WASO^e^ (17 min) Sensitive mode vs PSG. Underestimated TST^e^ (86 min) and SE^e^ (16.0%); overestimated SOL^f^ (12 min) and WASO^e^ (75 min); accuracy, 0.78 (SD 0.08); ﻿sensitivity, 0.78 (SD 0.09); specificity, 0.80 (SD 0.17) Sensitive mode vs actigraphy. Underestimated TST^e^ (127 min) and SE^e^ (22.9%); overestimated SOL^e^ (25 min) and WASO^e^ (102 min)
Brazendale et al (2019) [23]	Charge HR	Sleep log & actigraphy	Fitbit correlated with both actigraphy (r=.48)^f^ and sleep log (r=.71)^f^ for measuring TST
Brooke et al (2017) [24]	Flex & Charge HR	Sleep log	Both Fitbit Flex (r=.68, MAPE^k^=8.80%) and Fitbit Charge HR (r=.58, MAPE=11.5%) correlated with sleep log in measuring TST^e^
Cook et al (2017) [25]	Flex	PSG & actigraphy	Normal mode vs PSG. Overestimated TST^e^ (46 min) and SE^e^ (8.1%); underestimated WASO^e^ (44 min) and ﻿SOL (2 min, NS); accuracy, 0.88 (SD 0.05); sensitivity, 0.98 (SD 0.02); specificity, 0.35 (SD 0.13) Normal mode vs actigraphy. Overestimated SOL^e^ (12 min), SE^f^ (1.1%), and TST (5 min, NS); underestimated WASO^e^ (17 min) ﻿Sensitive mode vs PSG. Underestimated TST^e^ (86 min) and SE^e^ (16.0%); overestimated SOL^f^ (12 min) and WASO^e^ (75 min); accuracy, 0.78 (SD 0.08); ﻿sensitivity, 0.78 (SD 0.09); specificity, 0.80 (SD 0.17) Sensitive mode vs actigraphy. Underestimated TST^e^ (127 min) and SE^e^ (22.9%); overestimated SOL^e^ (25 min) and WASO^e^ (102 min)
Cook et al (2019) [26]^b^	Alta HR	PSG	Overestimated TST^f^ (12 min), SE^f^ (2.0%), and deep sleep^e^ (18 min); underestimated SOL (4 min, NS), WASO (8 min, NS), and light sleep (11 min, NS); accuracy, 0.90 (SD 0.04); sensitivity, 0.96 (SD 0.02); specificity, 0.58 (SD 0.16); accuracy in detecting light sleep, 0.73; deep sleep, 0.89; REM^l^ sleep, 0.89
de Zambotti et al (2016) [27]	Charge HR	PSG	Overestimated TST^f^ (8 min) and SE^e^ (1.8%); underestimated WASO^f^ (6 min) and SOL (3 min, NS); accuracy, 0.91 (SD 0.05); sensitivity, 0.97 (SD 0.02); specificity, 0.42 (SD 0.16); predictive value for sleep, 0.93 (SD 0.05); predictive value for wake, 0.65 (SD 0.18)
de Zambotti et al (2018) [28]^b^	Charge 2	PSG	Normal sleeper cohort. Overestimated TST^f^ (9 min) and light sleep^e^ (34 min); underestimated SOL^f^ (4 min), deep sleep^e^ (24 min), WASO (5 min, NS), and REM sleep (1 min, NS); sensitivity, 0.96; specificity, 0.61; accuracy in detecting light sleep, 0.81; deep sleep, 0.49; REM sleep, 0.74 PLMS^j^ cohort. Underestimated deep sleep^f^ (28 min), SOL (7 min, NS), and WASO (1 min, NS); overestimated TST (8 min, NS), light sleep (36 min, NS), and REM sleep (0 min, NS); specificity, 0.62; accuracy detecting light sleep, 0.78; deep sleep, 0.36; REM sleep, 0.62
Dickinson et al (2016) [29]	Charge HR	Actigraphy	No systematic difference across days between Fitbit and actigraphy in measuring TST and SE
Hakim et al (2018) [30]	Charge HR	PSG	Overestimated TST^f^ (30 min); underestimated total wake time^f^ (23 min)
Kang et al (2017) [31]	Flex	Unattended PSG	Good sleepers—normal mode. Overestimated TST^f^ (7 min), SE (1.8%, NS), and SOL (1 min, NS); underestimated WASO (7 min, NS); accuracy, 0.93; ﻿sensitivity, 0.97; specificity, 0.36 Insomniacs—normal mode. Overestimated TST^e^ (33 min) and SE^e^ (7.9%); underestimated WASO^e^ (31 min) and SOL (2.4%, NS); accuracy, 0.87; ﻿sensitivity, 0.97; specificity, 0.36 Good sleepers—sensitive mode. Accuracy, 0.66; ﻿sensitivity, 0.65; specificity, 0.82 Insomniacs—sensitive mode. Accuracy, 0.68; ﻿sensitivity, 0.64; specificity, 0.89
Kubala et al (2019) [32]	Alta	Actigraphy	Good sleepers. Overestimated TST^e^ (74 min); underestimated WASO^f^ (16 min) Poor sleepers. Overestimated TST (20 min, NS); underestimated WASO (13 min, NS)
Lee et al (2017) [33]	Charge HR	Actigraphy	Overestimated TST^e^ (22 min); correlation between Fitbit and actigraphy: sleep start times^e^ (r=.87) and TST^e^ (r=.92)
Lee et al (2018) [34]	Charge HR	Sleep log	Correlation between Fitbit and sleep log: TST^e^ (r=.55, MAPE 14.2%) and TIB^e,n^ (r=.48, MAPE 12.7%); SE and WASO not correlated
Liang and Chapa Martell (2018) [35]	Charge 2	Sleep Scope (EEG^o^ based)	Overestimated WASO^e^ (25 min) and deep sleep^e^ (40 min); underestimated TST^f^ (12 min), SOL^e^ (11 min), REM^e^ sleep (12 min), light sleep^e^ (42 min), and SE (1.5%, NS)
Liu et al (2019) [36]^b^	Alta HR	Sleep log	Overestimated WASO^f^ (13 min); underestimated TST^f^ (6 min), SOL^f^ (5 min), and SE^f^ (1.4%)
Mantua et al (2016) [37]	Flex	PSG (ambulatory system)	No significant difference in measuring TST and SE; TST correlated^e^ (r=.97); SE not correlated (r=.21, NS); average percentage error: TST, 2.97%; SE, 11.57%
Maskevich et al (2017) [38]	One	PSG	Overestimated TST^e^ (88 min) and SE^e^ (17.4%); underestimated WASO^f^ (39 min) and SOL (17 min, NS); accuracy, 0.81 (0.68-0.93); sensitivity, 0.99 (0.97-1.00); specificity, 0.27 (0.12-0.55); predictive value for sleep, 0.99, and wake, 0.27
Meltzer et al (2015) [39]	Ultra	PSG & actigraphy (2 different ones)	Fitbit—normal mode vs PSG. Underestimated WASO^e^ (32 min); overestimated TST^e^ (41 min) and SE^e^ (8%); accuracy, 0.84; ﻿sensitivity, 0.87; specificity, 0.52 Fitbit—sensitive mode vs PSG. Underestimated TST^e^ (105 min) and SE^e^ (21%); overestimated WASO^e^ (106 min); accuracy, 0.71; ﻿sensitivity, 0.70; specificity, 0.79
Montgomery-Downs et al (2012) [40]	Classic	PSG & actigraphy	Fitbit vs PSG. Overestimated SE^e^ (14.5%) and TST^e^ (67 min); sensitivity, 0.98 (0.92-1.00); specificity, 0.20 (0.02-0.78) Fitbit vs actigraphy. Overestimated SE^e^ (5.2%) and TST^e^ (24 min)
Osterbauer et al (2016) [41]	Flex	PSG	TST by Fitbit and PSG correlated^f^ (rho^p^=.99); WASO, SE, and awake minutes not correlated; sensitivity, 0.99; specificity, 0.10
Sargent et al (2018) [42]	Charge HR	PSG	TST by Fitbit vs PSG: NS; Fitbit automatically identified 60% of sleep periods, with a success rate of 80% when sleep was 9h, 90% when sleep was 8h, 70% when sleep was 7h, 50% when sleep was 2h, and 10% when sleep was 1h
Svensson et al (2019) [43]^b^	Versa	Sleep Scope (EEG based)	Overestimated TIB (9 min, NS), TST (7 min, NS), WASO^e^ (14 min), and deep sleep^e^ (36 min); underestimated SE (0.1%, NS), SOL^e^ (14 min), REM^e^ sleep (6 min), and light sleep^e^ (20 min); accuracy, 0.89 (0.88-0.89); sensitivity, 0.92 (0.919-0.923); specificity, 0.54 (0.53-0.55)

^a^Accuracy, sensitivity, and specificity in detecting sleep epochs are reported unless otherwise specified.

^b^Publication consisting of newer-generation, sleep-staging Fitbit models.

^c^PSG: polysomnography.

^d^TST: total sleep time.

^e^*P*<.01.

^f^*P*<.05.

^g^SE: sleep efficiency.

^h^WASO: wake after sleep onset.

^i^SOL: sleep onset latency.

^j^NS: not significant.

^k^MAPE: mean absolute percent error.

^l^REM: rapid eye movement.

^m^PLMS: periodic limb movement in sleep.

^n^TIB: time in bed.

^o^EEG: electroencephalographic.

^p^Spearman correlation coefficient.

### Bias Assessment

Table S1 in Multimedia Appendix 1 summarizes the risk for bias of research methods, internal validity, reported outcomes, and generalizability of each qualifying study. Only 5 investigations out of 22 (23%) attempted to blind the sleep laboratory technicians from outcome measures [22,25,26,28,40]. A total of 13 of the qualifying 22 studies (59%) relied on PSG as reference to evaluate Fitbit performance [22,25-28,30,31,37-42]; the other 9 (41%) relied on a sleep log, actigraphy, or home EEG as reference. Since PSG is the *gold standard* for measurement of sleep stages and parameters, use of other methods of reference constitutes an additional potential source of bias. Half of the investigations (11/22, 50%) were performed in the participant’s home. Clock hour of bedtime was unspecified in 5 of the 22 studies (23%), and the dictated bedtime differed from the habitual one of participants in 2 laboratory studies (9%). Disparity between habitual versus mandated bedtime might have resulted in a greater- or lesser-than-usual amount of wake time while in bed and this is likely to have led to bias. Moreover, use of somewhat bulky sleep trackers as reference for studies conducted in one’s residence might be problematic because of improper donning of instrumentation and oversight by technicians. Most studies (16/22, 73%) followed the manufacturer’s recommendation that the Fitbit model be worn on the nondominant hand. However, 2 out of the 22 studies (9%) utilized a counterbalance experimental design for placement of the Fitbit device, with half of the participants wearing it on the right hand and the other half wearing it on the left hand. Finally, in 2 out of the 22 studies (9%), the Fitbit was worn on the left wrist; in 2 other studies (9%), the hand upon which the Fitbit was worn was unreported. Out of the 22 studies, 1 (5%) [28] did not report the SE for Fitbit but did report it for PSG; this may constitute selective reporting bias. We contacted the corresponding author of this investigation for the missing information, but we did not obtain a response.

### Comparison of Sleep Parameters Assessed by Fitbit Versus Polysomnography

#### Nonsleep-Staging Fitbit Models

Out of the 22 studies, 10 (45%) assessed early-generation nonsleep-staging Fitbit models in comparison with PSG in estimating sleep parameters [25,27,30,31,37-42]; 1 of these studies involved performance of Fitbit models when applied to individuals of two different cohorts (ie, good sleepers and insomniacs), thereby increasing the number of possible comparisons to 11. Eight (N=203) of the 10 potential comparisons reported significant overestimation of TST by Fitbit versus PSG of between 6.5 and 88.1 minutes, while the two others (N=52) found nonsignificant overestimation. Five (N=142) of the six potential comparisons reported significant underestimation of WASO by Fitbit versus PSG of between 5.6 and 44 minutes, while one other (N=17) reported nonsignificant underestimation. Six (N=166) of the eight potential comparisons observed significant overestimation of SE by Fitbit versus PSG of between 1.8% and 17.4%, while two others (N=57) reported nonsignificant overestimation. A total of 5 comparisons (N=110) evaluated SOL, finding no significant difference between the two methods of appraisal.

**Figure 2:**
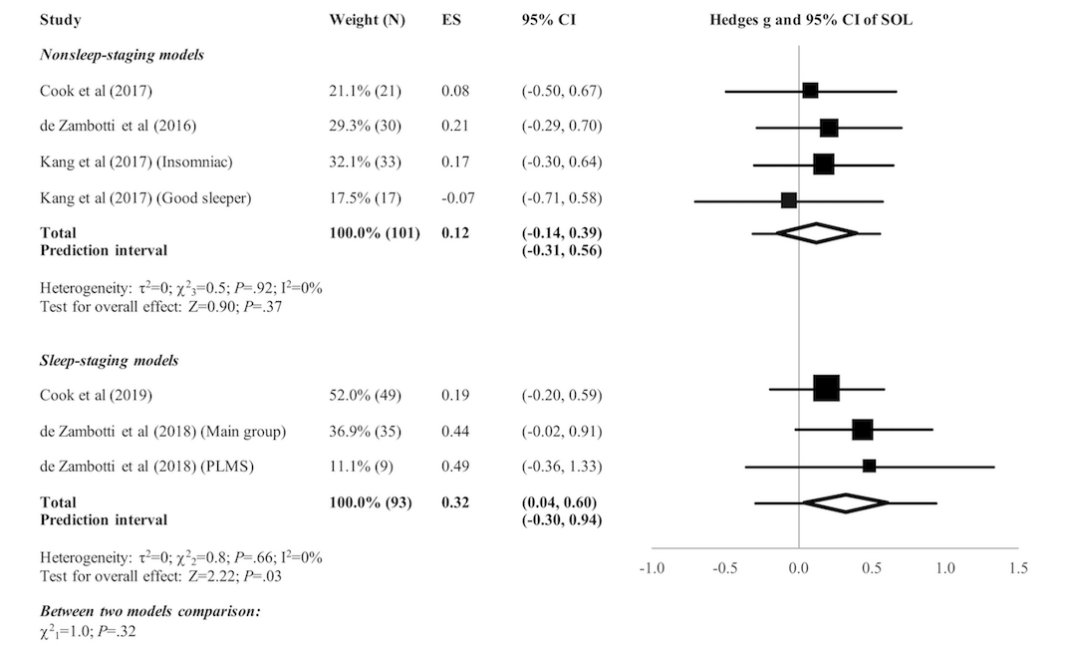
Forest plot of the standardized mean difference (Hedges g) between Fitbit and polysomnography for the variable of sleep onset latency (SOL). Results are shown as effect size (ES) and 95% CI. The difference in symbol size indicates the difference in weight of the respective studies. The diamond symbol shows the 95% CI of the overall effect and the tails show the 95% prediction interval of the overall effect. PLMS: periodic limb movement in sleep.

**Figure 3:**
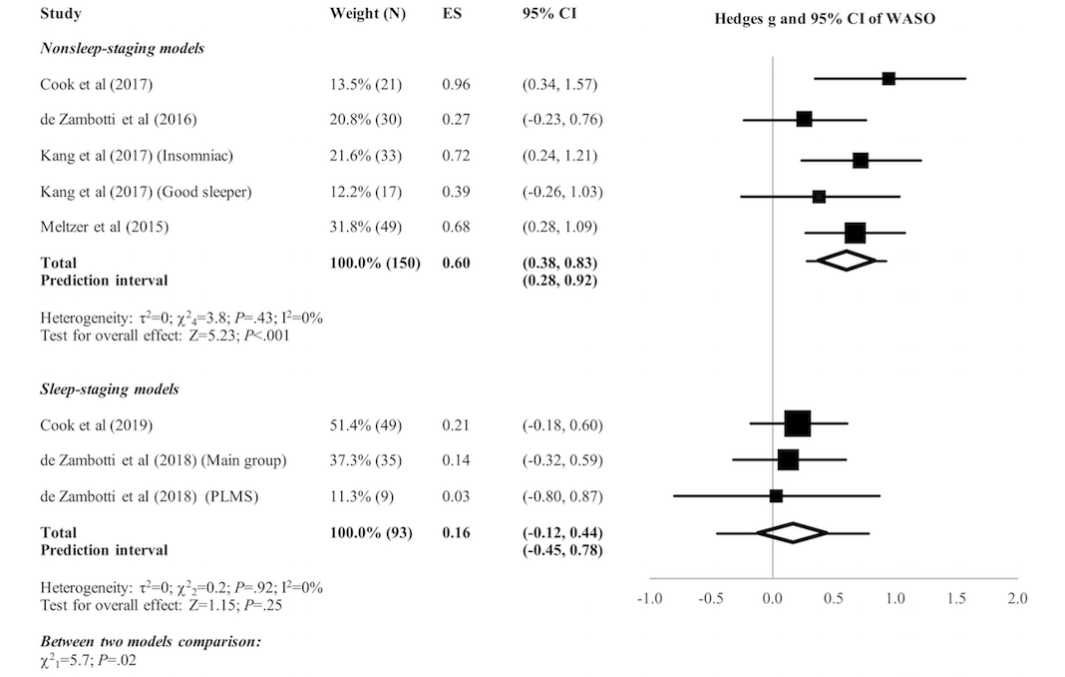
Forest plot of the standardized mean difference (Hedges g) between Fitbit and polysomnography for the variable of wake after sleep onset (WASO). Results are shown as effect size (ES) and 95% CI. The difference in symbol size indicates the difference in the weight of the respective studies. The diamond symbol shows the 95% CI of the overall effect and the tails show the 95% prediction interval of the overall effect. PLMS: periodic limb movement in sleep.

**Figure 4:**
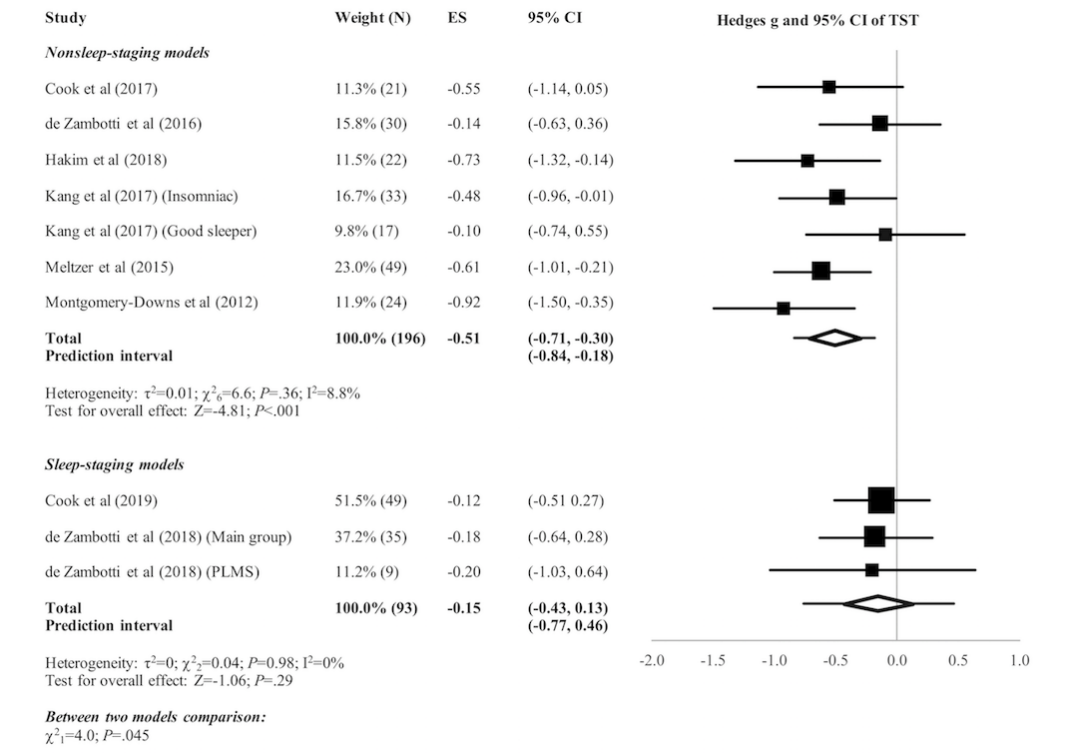
Forest plot of the standardized mean difference (Hedges g) between Fitbit and polysomnography for the variable of total sleep time (TST). Results are shown as effect size (ES) and 95% CI. The difference in symbol size indicates the difference in weight of the respective studies. The diamond symbol shows the 95% CI of the overall effect and the tails show the 95% prediction interval of the overall effect. PLMS: periodic limb movement in sleep.

**Figure 5:**
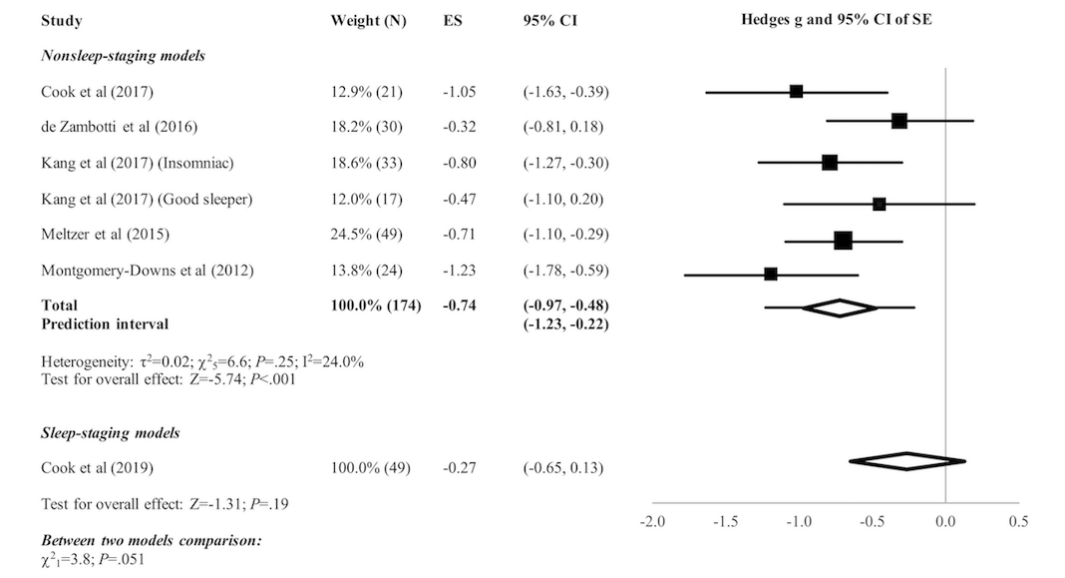
Forest plot of the standardized mean difference (Hedges g) between Fitbit and polysomnography for the variable of sleep efficiency (SE). Results are shown as effect size (ES) and 95% CI. The difference in symbol size indicates the difference in weight of the respective studies. The diamond symbol shows the 95% CI of the overall effect and the tails show the 95% prediction interval of the overall effect.

Figures 2-5 present the forest plots of effect size plus the overall pooled estimated effect size of the SOL, WASO, TST, and SE variables derived by nonsleep-staging Fitbit models. The pooled estimate of effect size reveals the following by nonsleep-staging Fitbit models relative to PSG: nonsignificant difference in estimation of SOL (N=4 comparisons; effect size=0.12, 95% CI -0.14 to 0.39; *P*=.37); significant underestimation of WASO (N=5 comparisons; effect size=0.60, 95% CI 0.38 to 0.83; *P*<.001); significant overestimation of TST (N=7 comparisons; effect size=-0.51, 95% CI -0.71 to -0.30; *P*<.001); and significant overestimation of SE (N=6 comparisons; effect size=-0.74, 95% CI -0.97 to -0.48; *P*<.001). Heterogeneity was not detected in any sleep parameter.

#### Sleep-Staging Fitbit Models

Only 3 publications out of 22 (14%) pertained to recent-generation sleep-staging Fitbit models in comparison with PSG as reference [22,26,28]; 1 of these 3 publications studied the performance of Fitbit on two different cohorts—normal and PLMS sleepers—thereby increasing the number of possible comparisons to four.

Two (N=84) of the three potential comparisons reported significant overestimation of TST by Fitbit versus PSG in the amount of 9-11.6 minutes, while one other (N=9) reported nonsignificant overestimation. The only study (N=49) that assessed SE reported 1.98% significant overestimation by Fitbit relative to PSG. A total of 3 trials (N=93) reported no significant difference in WASO between the two methods; 1 of these (N=35) found significant 4-minute underestimation of SOL by Fitbit versus PSG, while the 2 others (N=58) detected nonsignificant underestimation of SOL by Fitbit.

The pooled estimate of effect size (see Figures 2-5) revealed a significant underestimation of SOL (N=3 comparisons; effect size=0.32, 95% CI 0.04 to 0.60; *P*=.03) and a nonsignificant difference in estimation of WASO (N=3 comparisons; effect size=0.16, 95% CI -0.12 to 0.44; *P*=.25), TST (N=3 comparisons; effect size=-0.15, 95% CI -0.43 to 0.13; *P*=.29), and SE (N=1 comparison; effect size=-0.27, 95% CI -0.65 to 0.13; *P*=.19) by sleep-staging Fitbit models versus PSG. Heterogeneity was not detected in any sleep parameter. Since only 1 study evaluated SE, testing for heterogeneity was not relevant.

#### Comparison of Nonsleep-Staging Versus Sleep-Staging Fitbit Models

Subgroup analyses revealed no significant difference between nonsleep-staging and sleep-staging models in estimating SOL (χ^2^_1_=1.0, *P*=.32). However, sleep-staging models performed better than nonsleep-staging models in estimating WASO (﻿χ^2^_1_=5.7, *P*=.02), TST (χ^2^_1_=4.0, *P*=.045), and SE (χ^2^_1_=3.8, *P*=.051, ie, below the cutoff of *P*=.10 recommended for statistical significance of subgroup comparisons).

### Accuracy, Sensitivity, and Specificity in Detecting Sleep Epochs by Fitbit Versus Polysomnography

#### Nonsleep-Staging Fitbit Models

A total of 7 studies (N=197) involved epoch-by-epoch (EBE) investigation of nonsleep-staging Fitbit models with reference to PSG in differentiating between sleep and wake state [25,27,31,38-41]. Out of these 7 studies, 1 consisted of two different samples, thereby increasing the total number of evaluations to 8. Across these trials, Fitbit versus PSG analyses identified sleep epochs with accuracy values between 0.81 and 0.93, sensitivity values between 0.87 and 0.99, and specificity values between 0.10 and 0.52.

#### Sleep-Staging Fitbit Models

##### Sleep Epoch Identification

A total of 3 studies (N=153) evaluated the performance of sleep-staging Fitbit models with PSG as reference in identifying sleep epochs through EBE analyses [22,26,28]. A total of 1 study included two different samples, thereby increasing the number of possible comparisons to four. Relative to PSG, detection of sleep epochs in three possible comparisons (N=144) revealed sensitivity values between 0.95 and 0.96; detection of sleep epochs in four comparisons (N=153) revealed specificity values between 0.58 and 0.69; and detection of sleep epochs, assessed in only a single study (N=49), revealed an accuracy of 0.90.

##### Sleep-Stage Identification

A total of 3 studies (N=153) appraised performance of sleep-staging Fitbit models in identifying sleep stages by means of EBE analysis [22,26,28]. Relative to PSG, accuracy varied between 0.69 and 0.81 in detecting light sleep (non-rapid eye movement [REM] 1 [N1] + non-REM 2 [N2]), between 0.36 and 0.89 in detecting deep sleep (non-REM 3 [N3]), and between 0.62 and 0.89 in detecting REM sleep.

### Evaluation of Sleep Parameters Estimated by Fitbit Models Versus Actigraphy

No published study assessed performance of sleep-staging Fitbit models relative to actigraphy in estimating sleep parameters. In contrast, 7 studies investigated the accuracy of nonsleep-staging Fitbit models relative to actigraphy [23,25,29,32,33,39,40]; 1 of them involved two different actigraph devices [39], and another 1 included two different samples: good sleepers and poor sleepers [32]. Among the total of seven potential comparisons, Fitbit significantly overestimated TST in five of them (N=84) by 24.1-74 minutes, while in two comparisons (N=31), it was nonsignificantly overestimated. In a total of four comparisons (N=69), Fitbit significantly overestimated SE by 1.1%-7.0%. Among a total of five comparisons, Fitbit significantly underestimated WASO in four of them (N=65) by 16-32 minutes, while in one (N=10), it was nonsignificantly underestimated. Only 1 study (N=21) evaluated SOL, finding an 11.5-minute significant overestimation by Fitbit.

Two studies [25,38] simultaneously compared in the same cohort a nonsleep-staging Fitbit model plus actigraph against laboratory PSG. Relative to PSG, both the actigraph and Fitbit overestimated TST: Fitbit in one study by 88 minutes and in the other by 46 minutes; actigraph in one study by 74 minutes and in the other by 40.6 minutes. They also overestimated SE: Fitbit in one study by 17.4% and in the other by 8.1%; actigraph in one study by 14.8% and in the other by 7%. However, they underestimated WASO: Fitbit in one study by 39 minutes and in the other by 44 minutes; actigraph in one study by 20 minutes and in the other by 27 minutes. Actigraphy showed less bias than Fitbit. On the other hand, in the same two studies, Fitbit performed better than actigraphy in measuring SOL relative to PSG as reference: Fitbit bias in one study of 17 minutes and 2 minutes in the other; actigraph bias in one study of 23 minutes and 14 minutes in the other. One of these studies also performed EBE analysis [25], finding approximately 1% higher sensitivity and accuracy in detecting sleep by Fitbit than by actigraphy relative to PSG.

### Correlation Between Sleep Parameters Assessed by Fitbit Versus Sleep Diary

A total of 3 studies investigated the extent of correlation between sleep parameters derived by a nonsleep-staging Fitbit model and a self-rated sleep diary [23,34]; 1 of them involved two different Fitbit models [24]. In the total of four potential comparisons (N=104), significant correlation, ranging between r=.55 and r=.71, was reported between TST measured by Fitbit versus sleep diary. A total of 1 study (N=38) found significant correlation between the two methods of sleep assessment for time in bed (TIB; r=.48) but poor correlation for WASO (r=.09) and SE (r=-.03). A total of 1 study (N=10) [36] investigated the extent of agreement between the sleep diary and sleep-staging Fitbit methods. It reported significant overestimation of WASO (13 minutes) and underestimation of SOL (5 minutes), TST (6 minutes), and SE (1.4%) by Fitbit in comparison to the sleep diary.

### Dominant Versus Nondominant Hand Comparison of Fitbit Sleep-Stage Classification Accuracy

Only 1 study (N=60) [22] explored differences in accuracy of sleep-stage classification when new-generation Fitbit models were simultaneously worn on the dominant and nondominant hand. No between-hand difference was found in estimating sleep stages.

### Effect of Selected Sensitivity Mode Setting on Estimation of Sleep Parameters

Early-generation nonsleep-staging, but not later-generation sleep-staging, Fitbit models allow the user to select either normal or sensitive mode to sense body movement to derive sleep parameters. A total of 3 studies evaluated, relative to PSG, performance of these Fitbit models when set to the sensitive mode [25,31,39]. In 2 of them, the sensitive mode significantly underestimated TST by 86.3 minutes (N=21) and 105 minutes (N=63), respectively, and underestimated SE by 16.0% (N=21) and 21% (N=63), respectively. The only study (N=21) that evaluated SOL found that the sensitive mode significantly overestimated it by 11.5 minutes. Across the 3 studies (N=134), differentiation of sleep from wake epochs by early-generation nonsleep-staging Fitbit models when set to the sensitive mode relative to PSG ranged from 0.66 to 0.78 in accuracy, from 0.64 to 0.78 in sensitivity, and from 0.79 to 0.89 in specificity.

### Interdevice Reliability

A total of 3 studies evaluated interdevice reliability of nonsleep-staging Fitbit models. In 1 of them, 3 participants wore two Fitbit Classic bands on the same wrist, finding through EBE comparisons high interdevice reliability (96.5%-99.1%) [40]. Another study with 7 participants who wore two Fitbit Ultra devices on the same wrist also substantiated essentially equivalent between-device performance in estimating TST and SE; findings for other sleep parameters were not reported [39]. Finally, the remaining study involving 10 participants who simultaneously wore two Fitbit Alta devices on the wrist of their nondominant arm found no significant difference between the two devices in measuring WASO, but slight, yet statistically significant, difference (approximately 6 minutes) between them in measuring TST [32].

## Discussion

### Principal Findings

The quality and uses of personal monitoring technology are rapidly advancing, offering the promise of extensive improvement in medical literacy and health. An area of high interest today to consumers and health professionals is sleep quality because of its recognized importance to daytime cognitive and physical performance. A number of wrist-worn devices enable tracking of sleep parameters and stages. Some of the most popular ones are marketed by Fitbit, Inc; performance of several of its nonsleep-staging and sleep-staging models have been evaluated against laboratory PSG, home sleep trackers, or other methods. The objective of this systematic review was to comprehensively evaluate the worthiness of these consumer wristband devices in assessing sleep.

PSG is regarded as the gold standard for assessment of sleep parameters and stages; in comparison to PSG, nonsleep-staging Fitbit models overestimate TST and SE, underestimate WASO, but determine SOL equally well. Moreover, the amount of bias in estimating TST, SE, and WASO by such wristband models is not negligible. EBE analyses demonstrate, in comparison to laboratory PSG, the high accuracy and sensitivity of this type of Fitbit model in detecting sleep; however, the analyses demonstrate only modest specificity.

In 2007, the American Academy of Sleep Medicine certified wrist actigraphy for at-home evaluation of sleep patterns of both healthy adults and patients with certain suspected sleep disorders [44]. However, several studies have found actigraphy used in conjunction with any one of the four popular interpretative algorithms [2] overestimates sleep duration; although its sensitivity in detecting sleep is high (ie, between 0.87 and 0.99), its specificity is low (ie, between 0.28 and 0.67) [3]. This is the case because actigraphy and its interpretative algorithms tend to score epochs of quiet wakefulness as sleep [45]. Studies that compared nonsleep-staging Fitbit models with actigraphy [23,25,29,32,33,39,40] revealed that Fitbit overestimates both TST and SE and underestimates WASO. Only a single study [25] assessed SOL relative to actigraphy; it found that Fitbit significantly overestimated this sleep parameter, on average, by 12 minutes. The single study [25] that performed EBE analyses on the same participants who were simultaneously outfitted with nonsleep-staging Fitbit and actigraph devices reported only a minor (ie, approximately 1% higher) difference in sensitivity and accuracy by Fitbit. In this regard, nonsleep-staging Fitbit models enable user selection of either normal or sensitive mode to measure body movement to derive sleep parameters. The recommended normal mode only scores *significant* body movements while in bed as awake time. In contrast, the sensitive mode interprets nearly all such movements as awake or restless sleep time [46]. Studies that evaluated Fitbit devices relative to PSG when the devices were set to the sensitive and normal modes reported that the sensitive mode, compared to the normal mode, gave rise to notably higher bias in estimating SOL, SE, WASO, and TST; the sensitive mode also gave rise to lower accuracy and sensitivity but higher specificity. These findings confirm the company’s recommendation that the normal mode setting be used in most instances.

Fitbit introduced its sleep-staging feature in 2017, which is now incorporated into the Fitbit Charge 2, Fitbit Charge 3, Fitbit Alta HR, Fitbit Versa, Fitbit Versa 2, Fitbit Blaze, and Fitbit Ionic models. This feature relies on a combined body movement and HRV algorithm to identify and estimate time spent in individual sleep stages [5]. The Fitbit interpretative proprietary sleep-staging algorithm was derived using machine learning methods (ie, linear discriminant classifier) applied to three types of parameters—motion, HRV, and respiratory rate, with the last two calculated from heartbeat data sensed by photoplethysmography—measured during a sleep study of 60 normal sleepers, 18-60 years of age (mean 34, SD 10). These three groups of parameters led to an initial set of 180 features, which, through the method of recursive feature elimination, was reduced to 54 features. ﻿Subsequently, heartbeat-derived features were compared to movement-derived features and found to be of approximately equal importance in the overall classification and discrimination of sleep versus wake epochs [22]. Based on discussion with Fitbit, Inc, the same core hardware technology and software algorithm have been incorporated into all sleep-staging Fitbit models since their introduction in 2017, thereby making feasible valid performance comparisons across the published studies.

Thus far, only 3 qualifying studies investigated the performance of the newer-generation Fitbit hardware- and software-coupled technology relative to PSG [22,26,28]. Of these, 1 evaluated the performance of Fitbit for two different—normal sleeper and PLMS—cohorts, thereby increasing the number of possible comparisons to four, depending on the specific sleep parameters evaluated in individual trials. Although these comparisons showed that recent-generation Fitbit models display only moderate accuracy in detecting sleep stages, they were much better at estimating TST, SE, and WASO than were early-generation, nonsleep-staging Fitbit models. Overall, the amount of bias in estimating the majority of these sleep parameters by new-generation Fitbit models in reference to PSG was clinically negligible: less than 12 minutes in the three comparisons in which TST was determined, 1.98% in the single comparison in which SE was assessed [26], and less than 7 minutes in the three comparisons in which SOL was measured. Meta-analysis of the published data also substantiated the lack of a statistically significant difference between sleep-staging Fitbit models and PSG in measuring WASO, TST, and SE, and with effect sizes of differences in the range of *small*, even for SOL. EBE analyses conducted on the data from the same four comparisons also revealed high sensitivity (ie, between 0.95 and 0.96) and specificity (ie, between 0.58 and 0.69) in detecting sleep. These four comparison trials involving sleep-staging Fitbit models disclosed much higher specificity in detecting sleep than all of the nonsleep-staging Fitbit model comparison trials that reported specificity in the range of 0.10-0.52. Moreover, three of these four comparisons found no significant difference in WASO between methods of assessment. In contrast, five comparisons consisting of nonsleep-staging Fitbit models reported significant underestimation of WASO by 5.6-44 minutes. Collectively, these findings imply the body movement and HRV sensor and software algorithm technology of recent-generation sleep-staging Fitbit models, in comparison to the early-generation ones, more accurately detects wake epochs during intended sleep. These are very promising results, since the major drawback of body activity and movement-based trackers, such as actigraphs, is overestimation of sleep time and poor sensitivity of detecting WASO (ie, poor differentiation of wake from sleep epochs during spans of quiet bedtime activity) [45,47].

A concern about commercially available personal monitors is intra- and interdevice reliability. A total of 3 investigations [32,39,40] addressed this matter and they all reported acceptable consistency. The sole longitudinal (ie, 4 consecutive nights) investigation [29] found no systematic internight difference in TST and SE between Fitbit and actigraphy used as reference. The authors of this study concluded that Fitbit can be a useful device to assess trends in sleep quality, even though absolute values of some sleep parameters might be biased [29]. Of these studies, 1 reported a statistically significant, but nonetheless clinically nonsignificant, interdevice difference (approximately 6 minutes) in measuring TST [32].

Subjective self-report survey and diary methods, even though popular means of assessing sleep due to their ease of use and low cost, are of limited value. Self-report survey approaches depend on recall, which can be biased, especially when not restricted to recent, single point-in-time experiences. Sleep diary methods depend less on memory, but reported information may not be sufficiently accurate because of poor awareness of certain events, such as number or frequency of nighttime awakenings and precise time of falling asleep [48]. Studies that compared Fitbit models with sleep diary methods found a significant correlation between the two approaches for TIB and TST. Because of their simplicity and affordability, sleep survey and diary approaches are generally favored over actigraphy; however, the relatively inexpensive objective method of Fitbit models, along with their ease of use and better estimation of most sleep variables, may render them more appealing.

The findings of this systematic review pertaining to both nonsleep-staging and sleep-staging Fitbit models are based on our recent comprehensive search of databases for relevant published articles, which was repeated three different times during a 15-month span. The included research studies have certain limitations (eg, more than half of them were performed in a laboratory rather than a home setting, and participants were mostly young or middle-aged normal sleepers). Moreover, nonsignificant findings of studies with small sample sizes might be the consequence of insufficient statistical power. Furthermore, only 5 of the 22 qualifying published investigations involved Fitbit sleep-staging models. An additional limitation is a lack of published information as to the extent that advances in Fitbit hardware and/or software technology explain the described disparity in performance between the company’s different generations of models in deriving sleep parameters and stages. Based on our dialogue with company representatives, this disparity is due collectively to advances in sensor technology; improved fidelity of data acquisition; and incorporation of heart rate, HRV, and body movement into its sleep-study-validated proprietary interpretative algorithm. In spite of these limitations, findings of this review indicate that the advanced body movement and HRV method of recent-generation Fitbit wristband models seems appropriate to derive suitable estimates of sleep parameters and time spent in sleep. The findings further suggest that such Fitbit models may be useful for conduct of population-based sleep research, which, in the past, typically relied heavily or entirely upon subjective methods.

### Conclusions

Fitbit models are marketed to the lay public to allow users to self-derive knowledge about their sleep quality, rather than as a substitute for standard clinical polysomnography. They appear useful for the study of the 24-hour sleep-wake pattern and for the determination of the duration, pattern, and quality of sleep, longitudinally, over many consecutive nights under normal living conditions. In this regard, individuals can benefit from information obtained by wristband trackers to improve sleep hygiene and sleep, itself. In certain cases, primary care and sleep specialists can at least gain superficial perspective on the sleep of patients. In spite of the fact that there has yet to be sufficient evaluation of recent-generation sleep-staging Fitbit models, findings of the few, thus far, published studies imply that their performance in differentiating wake from sleep epochs is better than that reported in the literature for actigraphy.

## Appendix

Multimedia Appendix 1 Bias assessment of included studies.

## Abbreviations

CINAHL: Cumulative Index to Nursing and Allied Health Literature
EBE: epoch-by-epoch
EEG: electroencephalographic
HRV: heart rate variability
MAPE: mean absolute percent error
N1: non-rapid eye movement 1
N2: non-rapid eye movement 2
N3: non-rapid eye movement 3
PLMS: periodic limb movement in sleep
PRISMA: Preferred Reporting Items for Systematic Reviews and Meta-Analyses
PSG: polysomnography
REM: rapid eye movement
SE: sleep efficiency
SOL: sleep onset latency
TIB: time in bed
TST: total sleep time
WASO: wake after sleep onset
