# Supplementary material for: Accuracy of Wristband Fitbit Models in Assessing Sleep: Systematic Review and Meta-Analysis
Source: J Med Internet Res. 2019 Nov 28;21(11):e16273. doi: 10.2196/16273 (PMC6908975; doi:10.2196/16273)
Supplement: Multimedia Appendix 1 [file jmir_v21i11e16273_app1.docx]

Table S1. Bias assessment of included studies^*^

| Study | Q1 | Q2 | Q3 | Q4 | Q5 | Q6 | Q7 | Q8 | Q9 | Q10 | Q11 | Q12 | Q13 | Q14 | Q15 | Q16 | Q17 | Q18 | Q19 |
| --- | --- | --- | --- | --- | --- | --- | --- | --- | --- | --- | --- | --- | --- | --- | --- | --- | --- | --- | --- |
| Beattie et al. (2017) | Y | N | Y | Y | Y | Y | NA | Y | Y | Y | N | N | Y | Y | Y | Y | N | Y | Y |
| Brazendale et al. (2019) | Y | Y | N | N | Y | N | Y | N | Y | NC | Y | Y | N | Y | N | Y | N | Y | Y |
| Brooke et al. (2017) | Y | Y | Y | Y | Y | Y | N | N | Y | Y | Y | Y | N | Y | N | Y | N | N | Y |
| Cook et al. (2017) | Y | Y | Y | Y | Y | Y | NA | Y | Y | Y | Y | N | Y | Y | Y | Y | N | Y | Y |
| Cook et al. (2019) | Y | Y | Y | Y | Y | Y | NA | Y | Y | Y | Y | N | Y | Y | Y | Y | N | Y | Y |
| de Zambotti et al. (2016) | Y | Y | Y | Y | Y | Y | Y | Y | Y | Y | Y | N | N | Y | Y | Y | N | Y | Y |
| de Zambotti et al. (2018) | Y | Y | Y | Y | Y | Y | NA | Y | Y | Y | Y | N | Y | Y | Y | Y | N | Y | Y |
| Dickinson et al. (2016) | Y | Y | N | N | Y | N | NA | N | Y | Y | Y | Y | N | Y | N | NC | N | N | Y |
| Hakim et al. (2018) | Y | Y | Y | N | N | Y | Y | Y | Y | Y | NC | N | N | Y | Y | NC | Y | Y | Y |
| Kang et al. (2017) | Y | Y | Y | Y | Y | Y | Y | Y | Y | Y | NC | Y | N | Y | Y | Y | N | Y | Y |
| Kubala et al. (2019) | Y | Y | Y | Y | Y | Y | Y | Y | Y | Y | Y | Y | N | Y | N | N | N | N | Y |
| Lee et al. (2017) | Y | Y | Y | N | Y | N | Y | Y | N | Y | Y | Y | N | Y | N | Y | N | N | Y |
| Lee et al. (2018) | Y | Y | Y | Y | Y | Y | Y | N | N | Y | Y | Y | N | Y | N | N | Y | N | Y |
| Liang and Chapa Martell (2018) | Y | Y | Y | Y | Y | Y | NA | N | N | Y | Y | Y | N | Y | N | Y | Y | N | Y |
| Liu et al. (2019) | Y | Y | N | N | Y | Y | N | Y | Y | NC | Y | Y | N | Y | N | Y | N | N | Y |
| Mantua et al. (2016) | Y | Y | Y | N | Y | Y | Y | N | N | Y | NC | Y | N | Y | Y | N | N | Y | Y |
| Maskevich et al. (2017) | Y | Y | Y | N | Y | N | NA | N | Y | Y | Y | N | N | Y | Y | Y | Y | Y | Y |
| Meltzer et al. (2015) | Y | Y | N | N | Y | Y | Y | N | Y | Y | Y | N | N | Y | Y | Y | N | Y | Y |
| Montgomery-Downs et al. (2012) | Y | Y | Y | N | Y | Y | NA | Y | Y | Y | NC | N | Y | Y | Y | Y | Y | Y | Y |
| Osterbauer et al. (2016) | Y | Y | Y | Y | Y | Y | NA | N | N | Y | NC | N | N | Y | Y | Y | N | Y | Y |
| Sargent et al. (2018) | Y | Y | Y | N | Y | N | NA | Y | N | Y | N | N | N | Y | Y | Y | N | Y | Y |
| Svensson et al. (2019) | Y | Y | Y | N | Y | Y | Y | Y | Y | Y | Y | Y | N | Y | N | Y | N | N | Y |

*Y: yes, N: no, NA: not applicable, NC: not clear

**Methods Bias**

Q1: Is the hypothesis/aim/objective of the study clearly stated?

Q2: Are primary outcome variables clearly described in the Introduction or Methods section? If first mentioned in the Results section, the question should be answered no.

Q3: Are the inclusion and exclusion criteria guiding participant selection explained?

Q4: Are demographic and other germane characteristics of the studied participants clearly described? (At least, age, gender, and BMI)

Q5: Are the study methods/procedures clearly described?

**Results - Bias**

Q6: Are the values of major findings of the study clearly described?

Q7: Were unsuccessful measurements due to participant dropout or device non-performance explained clearly?

Q8: Except when the probability value is <0.001, are actual probability values reported (e.g. 0.035 rather than <0.05) for the main outcomes?

Q9: Does the study provide estimates of the random variability in the data of the outcome variables as inter-quartile range, standard error/standard deviation, or confidence intervals?

**External validity - Bias**

All the following criteria attempt to address the representativeness of the findings of the study and whether they may be generalized to the population from which the study participants were derived.

Q10: Were those participants who were prepared to participate representative of the entire population from which they were recruited?

Q11: Does the timing of experiment represent the habitual sleep time of participants?

Q12: Does the location of experiment represent the habitual sleep location of participants?

**Internal validity- Bias**

Q13: Was an attempt made to blind those measuring the main outcomes of the intervention?

Q14: Were the statistical tests used to assess the main outcomes appropriate?

Q15: Did investigators use PSG as reference?

Q16: Were the wearable devices positioned and utilized in a same way for all participants?

Q17: Did the investigators calculate and report effect size or power?

Q18: Was participant compliance reliable? If the instrumentation was not applied by the researcher, answer No.

Q19: Does the data support the final conclusion?
